# Childhood trauma and cognitive biases associated with psychosis: A systematic review and meta-analysis

**DOI:** 10.1371/journal.pone.0246948

**Published:** 2021-02-25

**Authors:** Jazz Croft, David Martin, Paul Madley-Dowd, Daniela Strelchuk, Jonathan Davies, Jon Heron, Christoph Teufel, Stanley Zammit

**Affiliations:** 1 Centre for Academic Mental Health, Population Health Sciences, Bristol Medical School, University of Bristol, Bristol, United Kingdom; 2 Cardiff University Brain Research Imaging Centre, School of Psychology, University of Cardiff, Cardiff, United Kingdom; 3 Division of Psychological Medicine and Clinical Neurosciences, Medical Research Council Centre for Neuropsychiatric Genetics and Genomics, Cardiff University School of Medicine, Cardiff, United Kingdom; University of Haifa, ISRAEL

## Abstract

Childhood trauma is associated with an increased risk of psychosis, but the mechanisms that mediate this relationship are unknown. Exposure to trauma has been hypothesised to lead to cognitive biases that might have causal effects on psychotic symptoms. The literature on whether childhood trauma is associated with psychosis-related cognitive biases has not been comprehensively reviewed. A systematic review and meta-analysis or narrative synthesis of studies examining the association between childhood trauma and the following biases: external locus of control (LOC), external attribution, probabilistic reasoning, source monitoring, top-down processing, and bias against disconfirmatory evidence. Studies were assessed for quality, and sources of heterogeneity were explored. We included 25 studies from 3,465 studies identified. Individuals exposed to childhood trauma reported a more external LOC (14 studies: SMD Median = 0.40, Interquartile range 0.07 to 0.52), consistent with a narrative synthesis of 11 other studies of LOC. There was substantial heterogeneity in the meta-analysis (I^2^ = 93%) not explained by study characteristics examined. Narrative syntheses for other biases showed weaker, or no evidence of association with trauma. The quality of included studies was generally low. Our review provides some evidence of an association between childhood trauma and a more external LOC, but not with the other biases examined. The low quality and paucity of studies for most of the cognitive biases examined highlights the need for more rigorous studies to determine which biases occur after trauma, and whether they mediate an effect of childhood trauma on psychosis.

## Introduction

Psychotic disorders such as schizophrenia are a leading cause of disability and contribute to an increasing global disease burden [1, 2]. Exposure to abuse and neglect during childhood is associated with an increased likelihood of psychosis across the spectrum of symptom severity from sub-clinical psychotic experiences to psychotic disorder [3]. Meta-analyses estimate that the risk of psychosis is increased by 2–3 times [4] in those exposed to childhood trauma. However, there is a paucity of knowledge about the mechanisms that might explain how exposure to childhood trauma leads to the development of psychotic experiences. Current treatments for psychosis are of limited efficacy [5], and there is therefore a pressing need to understand more about the aetiology of these disorders to identify targets for preventative interventions.

Cognitive (information-processing) biases have been posited as a mechanism by which exposure to trauma can lead to the development of psychotic phenomena such as hallucinations and delusional beliefs, and can be broadly categorised as biases in causal attribution, interpretation, and inference. Causal attribution refers to how individuals interpret the outcome of events in terms of agency and responsibility. Previous studies have found that people with psychosis are more likely to believe that external forces (e.g. luck, fate) are accountable for the outcome of events (external locus of control [6, 7]) and to ascribe causality to situational factors (e.g. other people, institutional bias) for these events (external attribution bias [8]) compared to people without psychosis. Biases in the interpretation of new information that have been associated with psychosis include a bias for identifying internally-generated information (e.g. thoughts or speech) as coming from an external source (e.g. media or other people; source monitoring bias [9]), an over-reliance on prior knowledge over incoming stimuli when interpreting new information (top-down processing bias [10]), and a bias for interpreting neutral stimuli as threatening (attention to threat bias [11, 12]). Finally, biases in inference include hasty decision making in probabilistic inference tasks (the ‘Jumping to Conclusions’ bias; JTC [13]), and a resistance to revising beliefs in light of new information (a ‘Bias Against Disconfirmatory Evidence’; BADE [14]) have also been reported in people with psychotic symptoms.

Several theoretical models of psychosis have posited cognitive biases as candidate mechanisms on the pathway from exposure to trauma to subsequent psychotic symptoms [15–18]. Perhaps the most comprehensive is the Bayesian Hierarchy model that integrates biological consequences of stress (such as dopaminergic and glutamatergic dysfunction) with cognitive neuroscience to describe how stressors could lead to biases in attribution, interpretation and inference through disruption in prediction error signalling, and how this can lead to the formation of both hallucinatory experiences and delusional beliefs [17, 19]. However, whilst the increased prevalence of cognitive biases in psychosis has been established and theoretical models of how trauma can lead to biases exist, it is not clear whether experiences of childhood trauma are associated with these same biases.

We sought to inform theoretical models of psychosis by systematically reviewing studies that have examined the association between exposure to childhood trauma and biases in cognition and perception. Our aim was to examine whether individuals who were, or were not, exposed to trauma before the age of 18 years differed in performance on tasks that assessed bias in the following domains: locus of control, attribution bias, source monitoring, probabilistic reasoning, bias against disconfirmatory evidence, and top-down processing.

## Methods

The full search protocol was pre-registered in Prospero (ID: CRD42017059401).

### Literature search

We (J.C.) searched the following databases: PsychInfo, OvidMedliner and PILOTs on 15^th^ February 2020 using relevant key words and subject headings for exposure to different types of trauma and for each specific cognitive and perceptual task (for a complete list see Search Terms in S1 File).

### Inclusion criteria

Inclusion criteria are detailed in the supplementary materials (see Protocol and Fig A in S1 File). Articles must have been published in a peer-reviewed journal, in English, and compared performance on cognitive or perceptual bias tasks (detailed below) between participants who reported exposure to trauma prior to 18 years of age and those who did not.

The biases included are described briefly below. It should be noted that whilst attention to threat biases are also commonly described in people with psychosis this bias was not included in our study as a review of research on trauma and attention to threat bias was recently published [20].

#### External locus of control

A locus of control (LOC) refers to the extent to which an individual believes themselves to be accountable for their actions and is a specific dimension of attributional style [21, 22].

#### External attribution bias

Attribution theory refers to the way in which an individual ascribes causality to events; either to personal qualities (internal) or to others or situational factors (external) [23, 24].

#### Source monitoring

Source Monitoring refers to an individual’s ability to track actions and speech as produced by themselves or others and is also referred to as reality monitoring [9, 25].

#### Jumping to conclusions bias

A ‘jumping to conclusions’ bias refers to individuals making judgements hastily based on limited information, which can lead to reaching unwarranted conclusions [13, 26].

#### Top-down processing bias

An overreliance on prior expectations when perceiving new stimuli, also referred to as a greater influence of ‘top-down’ modulation in visual and auditory domains [27, 28]. This has been observed in visual and auditory domains [10, 29].

#### Bias against disconfirmatory evidence

A bias against disconfirmatory evidence (BADE) refers to a bias against revising initial probability estimations when presented with additional evidence that may contradict an individual’s initial estimation [30, 31].

### Study selection and data extraction

One author (JC) screened all abstracts and obtained full texts of papers that might potentially meet inclusion criteria. Working independently, two authors (J.C and one of P.M.D, D.S, or J.D) screened full-text articles to determine if they met inclusion criteria (see Fig A in S1 File). Data were extracted independently (J.C & D.M). Any discrepancies in decisions at any stage of the screening were resolved by a third reviewer.

### Quality assessment

Internal validity was assessed by two authors (J.C & D.M) independently rated each study using a version of the Newcastle-Ottawa scale, a widely-used risk of bias tool for observational studies, adapted for this study by the study authors (see Fig B in S1 File). Studies were rated based on the presence or absence of the following criteria: i) Random or complete sampling (1 point), ii) Response rate of 75% or more (1 points), iii) Non-exposed sample representative of exposed sample (1 point), iv) Adjustment for confounders (max. 2 points), v) Observer bias minimised (1 point). Total scores were calculated by summing scores across these 5 criteria (possible score 0–6). We focused particularly on sampling strategy, observer bias, and adjustment for confounding as the most likely sources of biased results. The variables identified *a priori* as the most likely potential confounders were: sex, markers of cognitive functioning (e.g. IQ), socio-economic status and age.

### Data analysis

A meta-analysis was only possible for studies that examined LOC. For each study, the sample *n*, mean LOC score and standard deviation of the exposed and non-exposed groups were used to derive a standardised mean difference (SMD) in LOC score. For studies that measured LOC in separate groups that were exposed to trauma, means and standard deviations were combined according to Cochrane guidelines [32]. A random effects meta-analysis of the SMD was conducted using the ‘metan’ [33] command in STATA version 15. Heterogeneity was assessed using the *I*^*2*^ statistic. Possible publication bias was assessed using an Egger’s regression test [34]. Meta-regression was used to assess whether likely sources of variation (study quality, recruitment sample, mean age, and sex distribution of sample) were associated with effect estimates and explained any heterogeneity. Where insufficient data were available to conduct a meta-analysis, studies were summarised using a narrative synthesis.

## Results

### Search results

**Fig 1:**
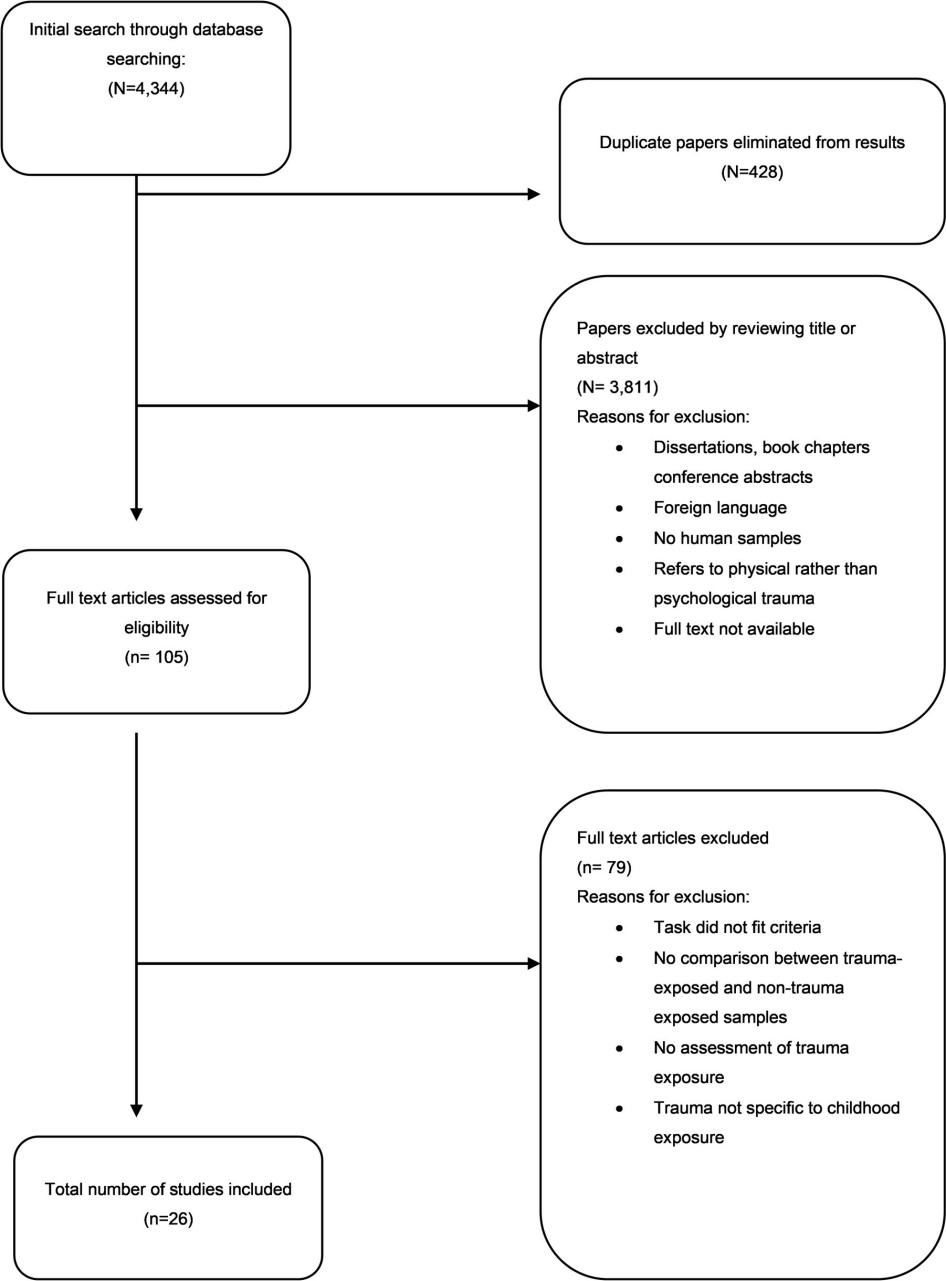
PRISMA flow chart. Flow chart of study selection.

The literature search resulted in a total of 4,144 references. After reading titles and abstracts of 3,906 de-duplicated results, 105 articles were reviewed in full and assessed for eligibility according to the inclusion criteria. After reading the full text, 79 full articles were excluded (PRISMA flow diagram, Fig 1).

### Included studies

We included 26 studies [21, 35–59] that fulfilled all search criteria (see Table 1 for summary of studies). The studies were based in the following countries: USA (12 studies) [21, 35, 38, 41, 43, 48, 50–55], UK (3 studies) [39, 44, 46], Australia (2 studies) [40, 49], and 1 each in Canada [47], China [59], Greece [36], Holland [45], Italy [56], Japan [58], New Zealand [57], Turkey [37] and Taiwan [42]. Six studies [38, 40, 48, 51, 52, 56] recruited separate exposed and unexposed samples and the remaining studies were cross-sectional designs. Thirteen studies recruited participants who were less than 18 years of age [37, 38, 43, 47–49, 51, 52, 55, 56, 58, 60], 12 recruited participants over 18 years of age [21, 35, 39, 40, 42, 44–46, 50, 53, 54, 57, 59] and 1 study [41] sampled participants across this age threshold. With the exemption of two studies that relied on assessments from health professionals [38, 56], studies obtained exposure data using a range of self-report questionnaires relating to different types of trauma (See Table A in S1 File). Twenty-six studies examined only one type of cognitive or perceptual bias. One study [48] examined both LOC and external attribution bias, contributing two results to the review. No included studies examined a bias for relying on top-down knowledge to disambiguate new information, or a bias against disconfirmatory evidence.

**Table 1:** Summary of studies included in the systematic review.

Study	Country	Sample Source	N	% female	Mean age (SD)	Trauma Type	N(%) exposed	Cognitive bias	Main Findings
Allen et al., 2017	USA	General population	4351	NR	33.2 (10.7)	Multiple	1789 (41)	LOC	Exposed: LOC = 30.31 (SD = 3.9)Unexposed: LOC = 30.16 (4.09); Reported as no difference (adjusted for sex)
Andreou, 2000	Greece	School	181	56	10.2 (1.7)	Bullying	34 (18.7)	LOC	Exposed: LOC = 12.13 (SD = 2.41) Unexposed: LOC = 7.23 (SD = 1.03)
Asberg et al., 2014	USA	Prison	39	100	37.82 (8.82)	Sexual Abuse	23 (59)	LOC	Exposed: LOC = 10.79 (SD 3.9) Unexposed: LOC = 8.87 (SD = 3.4) p = 0.15
Atik et al 2013	Turkey	School	742	53	13.11 (0.92)	Bullying	158 (21.3)	LOC	Exposed: LOC = 15.01 (SD = 4.38) Unexposed: LOC = 12.9 (SD = 4.40)
Barahal, 1981	USA	Social services & Non-exposed from local summer camp	33	31	7.5 (NR)	Multiple	17 (53)	LOC	Exposed LOC^1^ = 6 (SD 2.21) Unexposed: LOC = 8.7 (SD 1.62) p = .006 (adjusted for IQ)
Beck -Sander et al, 1997	UK	Outpatient	42	0	NR	Sexual Abuse, Physical Abuse	SA: 22 (52)	LOC	Exposed: LOC = 21.24 (SD = 18.5)
							PA: 21 (50)		Unexposed: LOC = 18.5 (SD = 5.8) Reported as NS
Bendall et al., 2011	AUS	Psychiatric centre	61	61	21.23 (2.47) - 22 (3.2)	Multiple	25 (40.3)	SM	Correlation reported as NS
Bolstad et al., 1997	USA	University	117	100	NR	Sexual Abuse	37 (31.6)	LOC	Exposed mean = 11.8 (SD = 3.9)
									Unexposed Mean = 12 (SD = 4.1)
									t(113) = .24 Reported NS
Chiu et al., 2016	Taiwan	Psychiatric Hospital	89	73	36 (12)	Multiple	NR	SM	Correlation reported as NS (adjusted for age, sex, years of education, psychopathology, global intellectual functioning)
Chiu et al., 2018	China	College Students	156	56	20.9 (1.3)	Multiple	45 (28.8)	SM	More errors in exposed group for experimenter-provided items (externally generated) than non-exposed group F(1, 154) = 10.34, p = .002. Source: Experimenter-provided = non-exposed M 0.84 SD 0.14 CI 0.81–0.87. exposed = M 0.75 SD 0.22 CI = 0.68–0.81
Fredstrom et al 2011	USA	School	695	56	15.84 (0.69).	Bullying	Technology: 193 (27.1)	LOC	Regression Technology-based: *b =* 0.84, SE = 0.14, p = < .001
									Regression of Technology and School Based in single model: Technology Based *b =* .63 SE = .14 p = < .001; School-based *b* = 0.69 SE = .14 p = < .001
							School-Based:NR		
Freeman et al 2008	UK	General Population	200	50	37.5 (13.3)	Multiple	NR	PRT	JTC bias present in 20% of sample OR = 1.1 (95% CI = .44, 2.75) p = .03
Hovens,et al 2016	Holland	Cohort Data Participants with baseline Depression and Anxiety	1,474	67	41.6 (12.3)	Multiple	846 (57.4)	LOC	Exposed LOC = 13.89 (SD = 6.67)
									Unexposed LOC = 12 (SD = 6.6)
									Beta coefficient for maltreatment score = 0.163 (p < .001) adjusted for sex, years of education and age
Ireland et al., 2015	UK	School	198	73	20.18	Sexual Abuse	44 (22.2)	LOC	Exposed = 46.1 (SD = 7.8)
									Unexposed = 48.2 (SD = 8.5)
									Analysis reported as NS
Luciano & Savage, 2007	Canada	School	27	48	10.9 (NR)	Bullying	NR	LOC	Correlation = .554 (p < .01)
									Adjusted for vocabulary and reading ability
Mannarino et al., 1996	USA	Rape crisis centre & matched controls	165	100	10 (NR)	Sexual Abuse	77 (46.7)	LOC; AS	LOC: Exposed = 16.6 (SD = 4.7)
									Unexposed = 15.7 (SD 4.9) t(1,164) = 1.1 NS; (Adjusted for ethnicity and SES)
									AS (bad events) Exposed = 7.4 (SD = 2.6) Unexposed = 6.4 (SD = 3.1) t(1,164) = 2.2 p < .05
Marsh et al., 2011	AUS	School	4,082	57	13.8 (1.4)	Bullying	NR	LOC	Positive relationship reported between external LOC and bully/victim factor loadings between .08-.26 p<05
McNally 2006	USA	General Population	174	73	NR	Bullying	138 (79.3)	SM	Sensitivity (d’), adjusted for sex:
									Block 1: r = 0.12, p = .07; block 2: r = 0.19, p = .01)
									No difference in response bias (criterion)
Moran & Eckenrode, 1992	USA	Social care & school	145	100	NR	Multiple	33 (22.8)	LOC	Mean LOC NR
									Multiple regression LOC (good events) B = .46, p = .01 AdjR2 = .14 B = .46, p = .01 (adjusted for age, parental SES, type of maltreatment)
Moyer at al	USA	Social care & school	201	100	NR	Sexual Abuse	43 (21)	LOC	Exposed = 16.7 (SE 0.66)
									Non-exposed = 12.2 (SE 0.38) p < .001
Muller 1994	USA	University	866	68	18.9	Physical Abuse	323 (36)	LOC	Exposed = 17.29 (SD = 4.9)
									Not Exposed 16.95 (SD = 4.83)
									Correlation = .21 p < .05
									(Adjusted for sex)
Porter & Long, 1999	USA	University	369	100	20 (3.98)	Sexual Abuse	84 (22)	LOC	Trauma = 12.81 (SD = 8.52)
									Not Exposed 11.28 (SD = 3.71)
									Reported NR (Adjusted for age)
Radliff et al 2016	USA	School	469	57	13.21, (0.97).	Bullying	277 (59)	LOC	Trauma = 14.33 (SD = 5.15) No Trauma 12.15 SD = 4.84) p = .003, Hedge’s g = .44, (Adjusted for school, age and grade)
Roazzi et al, 2016	Italy	Social Services referrals & General Population	160	37	10.96 (2.9)	Multiple	60 (37.5)	LOC	higher scores in maltreatment group on LOC (M = = 21.93 vs 18.56 F(1,152) = 14.84, p < .001. (Adjusted for SES).
Rucklidge, 2006	NZ	General Population	114	50	40.5 (12.2) - 44.8 (7.2)	Multiple	64 (57)	AS	Analysis reported as NS (Adjusted for age)
Yamasaki et al 2016	Japan	General population	4277	47	9.8 (0.4)	Bullying	522 (12.2)	LOC	4.66 (SD 1.89) Direct path coefficient Bullying—external locus of control .12 (p < .001)

NOTE abbreviations: JTC = ‘Jumping to Conclusions’ Bias PRT = Probabilistic Reasoning Task AS = Attribution Style NR = Not Reported NS = Not Significant ^1^ LOC measure reverse scored in analysis (a higher value denotes a less external LOC) * = combined groups: bully/victims and victims of bullying **combined mild and severe bullying. All LOC and AS scores are reported mean values and a higher LOC signifies a more external LOC and higher AS signifies more external AS.

### Association between trauma and bias

#### Locus of control

There were 20 studies that examined LOC. Nine different scales were used to assess LOC, the most common being the Nowicki-Strickland Scale for Children [61] (8 studies) [36, 37, 47, 48, 52, 55, 56, 58].^.^ Of the 20 studies, 14 had data available to compare the SMD in LOC scores between those exposed and unexposed to childhood trauma using a meta-analysis.

A total of 12,691 participants were included in the meta-analysis from these 14 studies with a median of 155 participants (range 27 to 4351). An average of 41.2% (SD = 18.7) of the participants were exposed to trauma. Results for sub-groups of trauma exposure were combined in a pooled analysis for three studies [36, 55, 62]. One study [39] reported two results from the same sample—one for exposure to physical abuse and one for exposure to sexual abuse; to reduce bias in the pooled analysis, only the exposure with the higher prevalence (sexual abuse) was included.

**Fig 2:**
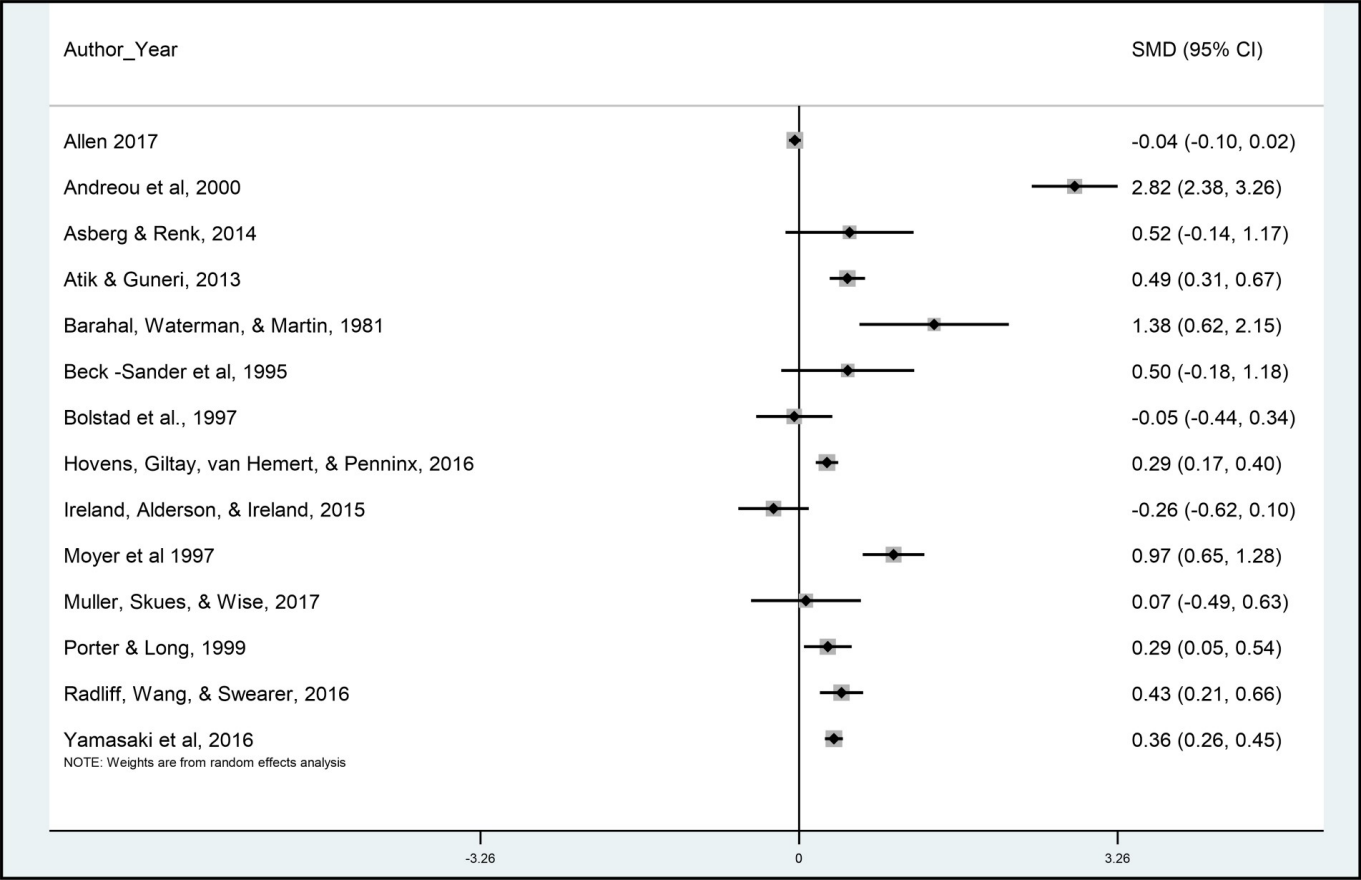
Meta-analysis forest plot of childhood trauma and Standardised Mean Difference (SMD) in locus of control.

The SMD suggested a greater (more external) LOC in the exposed group (SMD Median = 0.40, IQR 0.07 to 0.52). However, there was substantial heterogeneity between studies (I^2^ = 94.4%; Tau-squared = 0.15). As illustrated in Fig 2, one study [36] was a clear outlier in the analysis. When omitting this study, the I^2^ value reduced to 81.2% and Tau-Squared to .05.

A series of meta-regressions were carried out to examine possible sources of variation between studies. None of the variables we examined (study quality, recruitment sample, mean age, and sex distribution of sample) were associated with effect sizes across the included studies. The I^2^ value was reduced to the greatest extent by including the sex distribution of the sample (76.3%; see Table B in S1 File).

Five studies were not included in the meta-analysis as insufficient data were published and were not provided by the study authors [43, 47, 49, 51, 56] when requested. There was some evidence of an association between exposure to trauma and an external LOC reported by all five studies. Three studies examined forms of peer victimisation (correlation with a more external LOC ranging from r = .23 to r = .55; p-values < .05 to .003) [43, 47, 49] and two reported a relationship between maltreatment and a more external LOC [51, 56].

#### External attribution bias

Two studies examined the association between childhood trauma and external attribution bias. One study [57] examined the external attribution bias in participants with and without attention deficit hyperactivity disorder but found no association with childhood trauma in either group. In the other study, sexually abused children reported a greater personal attribution to negative events compared to non-abused children (p < .05) [48].

#### Source monitoring

Four studies examined performance on source monitoring tasks. Two of these did not report any evidence of an association between trauma exposure (62%-79% exposed) and source monitoring: one involved a sample of first-episode psychosis patients [40], and the other involved a sample of female, acute psychiatric patients [42]. In the third study, using a non-clinical sample, participants who reported exposure to sexual abuse had a lower sensitivity (d’) in distinguishing between real and imagined stimuli compared to non-exposed individuals (block 1: r = 0.12, p = .07; block 2: r = 0.19, p = .01) [50]. In the fourth study which was in a sample of university students, there was some evidence to suggest that participants exposed to trauma (29%) were more likely to misattribute externally-generated stimuli to self-generated sources in a hierarchical regression analysis after including confounders (B = -0.30, SE = 0.11, T = -2.86, p<0.01) [59].

#### Jumping to conclusions bias

Only one study [44] tested for the presence of the JTC bias, in a sample of 200 members of the general population. Twenty percent of this sample demonstrated this bias, but there was no association with childhood trauma (OR = 1.1, 95% CI 0.44, 2.75; p = .831). It should be noted that the proportion of the sample exposed to childhood trauma was not reported.

### Quality assessment

The assessment of quality for each paper is summarised in Table 2. Quality scores ranged from 2 to 6. Only six studies (23%) fulfilled over half of the criteria and the mean score across the 26 studies was 2.74 (SD = 1.02). The most poorly met criteria were related to sampling. Twenty-three studies (88%) did not use a random sample or sample a complete group, and 18 studies (69%) either had a low response rate (<75%) or failed to report a response rate. Of the 6 studies that described sampling from separate groups, 2 studies [63, 64] described sampling from the same community and were assessed as being representative of the exposed cohort. Of the remaining 4 studies, 2 [48, 65] reported a higher SES in the non-exposed group and adjusted for this in the analysis, and 2 [40, 65] did not provide details of whether the groups were from the same community.

**Table 2:** Quality assessment of included studies.

	Random/ complete sampling	Response rate ≥75%	Non-exposed representative of exposed	Adjusted for confounders^1^	Observer bias minimised	Total score
Allen et al., 2017	Yes	Yes	Yes	Yes*	Yes	5
Andreou, 2000	No	No	Yes	No	Yes	2
Asberg & Renk, 2014	No	No	Yes	No	Yes	2
Atik & Guneri, 2013	No	No	Yes	Yes*	Yes	3
Barahal, et al 1981	No	No	Yes	Yes*	No	2
Beck -Sander et al, 1997	Yes	Yes	Yes	No	Yes	4
Bendall et al., 2011	No	Yes	No	No	No	1
Bolstad et al., 1997	No	No	Yes	No	Yes	2
Chiu et al., 2016	No	Yes	Yes	Yes**	No	4
Chiu et al., 2018	No	No	No	Yes**	No	2
Fredstrom, et al., 2011	No	Yes	Yes	Yes*	Yes	4
Freeman, et al, 2008	No	No	Yes	No	Yes	2
Hovens, et al 2016	No	Yes	Yes	Yes*	No	4
Ireland, et al., 2015	No	No	Yes	No	Yes	2
Luciano & Savage, 2007	No	No	Yes	Yes*	No	2
Mannarino et al., 1996	No	No	No	Yes*	No	1
Mcnally et al., 2006	No	Yes	Yes	Yes*	Yes	4
Marsh et al., 2011	Yes	No	Yes	No	No	2
Moran et al., 1992	No	No	No	Yes**	No	2
Moyer at al., 1997	No	Yes	No	No	No	2
Muller 1994	No	No	Yes	Yes*	Yes	3
Porter & Long, 1999	No	No	Yes	Yes*	Yes	3
Radliff, et al., 2016	No	No	Yes	Yes *	Yes	3
Roazzi et al, 2016	No	No	Yes	Yes*	Yes	2
Rucklidge, 2006	No	-	Yes	Yes*	Yes	3
Yamasaki et al, 2016	Yes	No	Yes	No	Yes	4

NOTE ^1^Scored based on how important the confounders are that study adjusts for * = adjusted for one confounder ** = Adjusted for two or more confounders.

Sixteen (62%) of the studies included in the review described procedures that aimed to minimise observer bias in assessing the outcome, most commonly through delivering self-report measures. Sixteen studies (62%) controlled for at least one variable that was identified by reviewers as a potentially important confounder. Only 3 studies (12%) controlled for multiple confounding variables. The results from the Egger’s regression provides weak evidence of an asymmetrical distribution in the funnel-plot of the meta-analysis. The estimated bias coefficient is 3.15 (95% CI = < .001, 7.03, p = 0.050; for funnel plot of distribution see Fig C in S1 File).

## Discussion

This review provides a comprehensive summary of research that examines the relationship between exposure to trauma in childhood and cognitive and perceptual biases associated with psychosis. There was some evidence of a difference in performance on cognitive tasks between those exposed and not exposed to childhood trauma. However, this was not observed for all the cognitive and perceptual tasks included in our search criteria. With the exception of LOC, a very small number of studies assessed the external attribution, source monitoring and probabilistic reasoning biases and no studies examined a bias for relying on top-down knowledge to disambiguate new information, or a bias against disconfirmatory evidence.

Furthermore, there was substantial heterogeneity (defined as an I^2^ >75%) in the meta-analysis of measures of LOC, making the estimated pooled effect size for this measure difficult to interpret. Heterogeneity was reduced when a low-quality study that was a clear outlier [36] was omitted from the meta-analysis. However, there was minimal evidence that any of the study characteristics we examined could account for this heterogeneity, and it remained substantial. Nevertheless, the findings from the meta-analysis, supported by those from the narrative synthesis, are consistent with a more external LOC in participants exposed to trauma. Increased externality of an individual’s locus of control is associated with a range of negative mental health outcomes [66], and has been often described in people with psychosis [23]. An external LOC has also been shown to mediate part of the association between childhood trauma and psychotic experiences [7]. Explanations for how trauma can lead to causal attributions and a more external LOC include psychological (for example by generating feelings of inferiority [67] and undermining estimations of self-efficacy) and biological (for example through disruption of dopaminergic and glutamatergic pathways) ones. Indeed, current aetiological models of psychosis attempt to integrate epidemiological (including trauma), biological, psychological and cognitive findings to explain how psychotic symptoms develop [17, 68].

We also observed some evidence to suggest that children exposed to sexual abuse had a bias towards more external attribution in negative situations [48], although this was not observed in children exposed to multiple traumas in another study [64]. Further exploration of the role of trauma type was limited by the small number of studies that examined this. Findings for source monitoring deficits and the JTC bias were also limited by the small number of studies examining these. Furthermore, we found no studies that examined exposure to trauma and top-down processing biases or bias against disconfirmatory evidence.

The quality assessment of studies highlighted potential sources of bias in a large proportion of the studies: only eight of the studies included (30%) satisfied more than half of the quality assessment criteria. A small proportion of studies (n = 8; 30%) reported a response rate of 75% or more, with even fewer studies (n = 4; 15%) reporting random or complete sampling, raising the likelihood that the findings from this review are influenced by selection bias. The role of confounding variables was also considered in the quality assessment. Sixteen (62%) of the studies adjusted for confounders in their analysis, but only one study [69] provided information on both unadjusted and adjusted results, meaning the extent to which confounding explains the association between childhood trauma and cognitive biases remains unclear.

A number of studies not included in this review as they met some, but not all of the inclusion criteria are summarised in Table B in S1 File. These studies reported a relationship between trauma and a more external LOC, which is consistent with the findings of this review. However, the one study that examined the JTC bias and external attribution bias did not find evidence of an association with trauma [70].

### Strengths and limitations

The review’s research questions benefit from an established theoretical framework based on the premise that cognitive biases are a mechanism on the causal pathway between childhood trauma and psychotic symptoms. A methodological strength was that we followed PRISMA guidelines throughout the review (see S2 File). The meta-analysis was based on data from a large number of participants and the majority of studies that tested LOC used scales that could be standardised for use in a pooled analysis. We were unable to include all studies in the meta-analysis because some studies did not provide the required data, either within the paper or on request. The review was limited by its restriction to English-language and peer-reviewed publications, so we may have missed studies that could have contributed to addressing our study aims.

As summarised in the supporting information (Table A in S1 File), there was a diverse range of measures used to assess trauma, including referrals from social services and various self-report measures, and this may have contributed to the wide range of exposure prevalence and the variation in results across these studies. Measurement error could also vary across measures as childhood trauma data collected by questionnaire may be less reliable compared to data collected by interview [71]. In addition, few studies distinguished between witnessed and experienced trauma, or provided information on other indicators of trauma severity that may have also contributed to heterogeneity in our findings. Furthermore, while we were able to test if the average age of the sample was a predictor of effect size, we were unable to examine whether the time since trauma exposure (recency effect) contributed to heterogeneity. Finally, while most studies measured LOC using well-established measures, including the Nowicki-Strickland and Rotter scales, studies that used less widely implemented scales may have increased variation in results across studies.

### Implications

Our study shows that there are very few studies that examine the relationship between exposure to childhood trauma and cognitive and perceptual biases associated with psychosis. The exceptions to this are LOC as reviewed here and attention to threat bias, as reviewed separately [20]. Results from analyses of these biases seem to support an association with trauma, though confidence that these associations are causal rather than due to confounding or bias is low. Based on these results, there is a clear need for future studies to employ more rigorous methodology and to examine the role of confounding more thoroughly. For example, markers of adverse family environments (such as poverty, parental substance use, and parental psychiatric disorder), as well as child-related factors such as temperament and cognitive ability could all be associated with risk of childhood trauma exposure and also affect the way a child perceives and makes inferences about the world around them. Clearer reporting of descriptive statistics and results of any tests of association, including both unadjusted and adjusted estimates, would also make it easier to combine estimates in future meta-analyses.

Our review highlights an important evidence gap in our understanding of potential mechanisms by which a greater risk of psychosis might arise in those exposed to childhood trauma. Further study is needed to ascertain whether the aforementioned information-processing biases occur as a result of trauma and the extent to which they mediate the relationship between trauma and psychosis. This might then help to identify those biases most likely to offer potential targets for the development of new therapeutic interventions for psychotic symptoms.

## Conclusions

Our review provides some evidence of an association between exposure to childhood trauma and a more external LOC, but not for the other cognitive biases examined here. Whilst an external LOC might be a candidate mechanism that mediates an effect of childhood trauma on psychosis, most studies that examined this relationship were of low quality, and further studies are required to strengthen the currently weak evidence that this bias arises as a consequence of trauma.

## Supporting information

S1 File Supplementary information. (DOCX)

S2 File PRISMA checklist. (DOCX)
