# Supplementary material for: Childhood trauma and cognitive biases associated with psychosis: A systematic review and meta-analysis
Source: PLoS One. 2021 Feb 25;16(2):e0246948. doi: 10.1371/journal.pone.0246948 (PMC7906349; doi:10.1371/journal.pone.0246948)
Supplement: S1 File — (DOCX) [file pone.0246948.s001.docx]

Contents

[Supplementary Information 1](#_Toc62657807)

[Protocol 2](#_Toc62657808)

[Search Terms 5](#_Toc62657809)

[Fig. A Screening Checklist 7](#_Toc62657810)

[Fig. B Adapted Newcastle-Ottawa Quality Assessment 8](#_Toc62657811)

[Fig. C Funnel plot of Egger’s Test Distribution with pseudo-95% confidence intervals for the association between Childhood Trauma and Locus of Control 9](#_Toc62657812)

[Table A Methods of Trauma Assessment in Studies Included in the Review 10](#_Toc62657813)

[Table B Meta-Regression of Pooled Analysis of Association Between Childhood Trauma and Locus Of Control 12](#_Toc62657814)

[Table C: Selected studies meeting some, but not all inclusion criteria 14](#_Toc62657815)

[Bibliography 15](#_Toc62657816)

# Protocol

**Review question(s)**

The relationship between trauma and cognitive and perceptual biases associated with psychosis: A systematic review

**Condition or domain being studied**

Cognitive and perceptual bias outcomes of CT.

**Participants/ population**

Can include participants from both clinical and non-clinical populations

**Inclusion criteria**

Studies must:

Have a study design which examines the relationship between a measure of exposure to traumatic events and a measure of a cognitive or perceptual bias associated with psychotic symptoms.

Have been published in a peer-reviewed journal

Include an analysis of the relationship between childhood trauma and a test of cognitive bias

Study design: can include any study design

Exclusion criteria:

Experimental designs where baseline levels of childhood trauma exposure and/or cognitive bias or perceptual bias are not recorded prior to interventions that aim to alter these biases will not be included

Not published in the English language.

**Condition or domain being studied**

The domains studied in the review are child trauma and cognitive and perceptual bias associated with psychosis. Childhood trauma is defined as exposure to traumatic events prior to 18 years old including physical abuse, emotional abuse, sexual abuse, bullying, emotional neglect and physical neglect.

Cognitive biases and perceptual biases that are associated with psychotic symptoms, which are defined as cognitive and perceptual distortions that have been found to support the development and maintenance of psychosis (van der Gaag et al., 2013).

**Intervention(s), exposure(s)**

Exposure to childhood trauma

**Comparator(s)/ control**

Participants that do not report childhood trauma

**Context**

Any setting. Both clinical and non-clinical populations can be included

**Outcome(s)**

**Primary outcomes**

Performance on tasks that test for cognitive biases associated with psychotic symptoms. This is defined as the following in these tasks:

The jumping-to-conclusions bias: This should either be the beads task (Huq et al., 1988) or a conceptual equivalent.

Externalizing bias: A task that tests for a bias for misattributing internal thoughts or spoken words to an external source (Brookwell et al., 2006)

Top-down processing: A task that tests for a top-down processing bias associated with hallucinations (Aleman et al. 2003; Seal, et al 2004)

External attribution bias: A task that tests for participants’ bias to attribute negative events to external sources (Kaney and Bentall, 1989; Sullivan et al., 2013)

Belief inflexibility Bias: A task that tests for a bias against disconfirmatory evidence (BADE; waetcher et al., 2014; Eisenacher & Zink, 2016)

Tasks that explicitly state that the outcome is a perceptual or cognitive bias associated with psychosis that is not one of the above mentioned tasks, or a conceptual equivalent, will be identified and not included in main analysis but discussed in final results

**Searches**

EMBASE, PILOTS, MEDLINE, MEDLINE-in-process, PSYCHinfo will be searched using the following terms:

Hand searches will be conducted of reference sections of eligible studies. Leading researchers in the area will also be contacted to provide any further resources.

Searches will be made using the following search terms:

Information processing: bias* or error or deficit or reason* and cognition or cognitive or meta-cog* or attention$ or process$ or perception or perceptual or information-processing or information adj processing

Jumping to Conclusions bias: JTC or Jumping adj2 conclusions or jump adj2 conclusion or data gathering or beads adj2 task or probab* reas*

Source Monitoring: source adj memory or source adj monitoring or reality adj monitoring or source adj recognition or self adj recognition or self adj monitoring or external adj monitoring or external misattribut* or self adj generated speech

Top-down processing: Top-down adj processing or top adj down adj processing or auditory adj feedback or visual adj feedback

Externalising Bias: cognit* styles or locus of control or attribut* style or external adj2 attribut* or attribut* style or externali$* bias or internal attribut*

Trauma: trauma* or maltreat* or abuse or advers* or neglect or bully* or victim


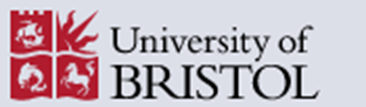

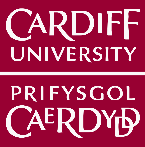
Belief inflexibility: belief inflex* or belief flex* or BADE or disconfirm* bias* or evidence integrat*

**Data extraction, (selection and coding)**

Titles and abstracts of studies retrieved using the search strategy and those from additional sources will be screened to identify studies that potentially meet the inclusion criteria outlined above. The full texts of these potentially eligible studies will then be retrieved and independently assessed for eligibility by two review team members. Any disagreements between them over the eligibility of particular studies will be resolved through discussion with a third reviewer.

**Risk of bias (quality) assessment**

A checklist will be used to assess the risk of bias including confounding variables, information bias, recruitment bias and reporting bias

**Strategy for data synthesis**

We will provide a narrative synthesis of the findings from the included studies.

**Analysis of subgroups or subsets**

None planned.

**Dissemination plans**

A paper will be submitted to a leading journal in this field.

**References**
Aleman, André, Koen B. E Böcker, Ron Hijman, Edward H. F de Haan, and René S Kahn 2003 Cognitive Basis of Hallucinations in Schizophrenia: Role of Top-down Information Processing. Schizophrenia Research 64(2–3): 175–185.

Bar-Haim, Y., Lamy, D., Pergamin, L., Bakermans-Kranenburg, M. J., & van IJzendoorn, M. H. (2007). Threat-related attentional bias in anxious and nonanxious individuals: A meta-analytic study. *Psychological Bulletin*, *133*(1), 1–24. https://doi.org/[10.1037/0033-2909.133.1.1](http://dx.doi.org/10.1037/0033-2909.133.1.1)

Brookwell, M. L., Bentall, R. P., & Varese, F. (2013). Externalizing biases and hallucinations in source-monitoring, self-monitoring and signal detection studies: a meta-analytic review. *Psychological Medicine*, *43*(12), 2465–2475. https://doi.org/[10.1017/S0033291712002760](http://dx.doi.org/10.1017/S0033291712002760)

Eisenacher, S., & Zink, M. (n.d.). Holding on to false beliefs: The bias against disconfirmatory evidence over the course of psychosis. *Journal of Behavior Therapy and Experimental Psychiatry*. https://doi.org/[10.1016/j.jbtep.2016.08.015](http://dx.doi.org/10.1016/j.jbtep.2016.08.015)

Huq, S. F., Garety, P. A., & Hemsley, D. R. (1988). Probabilistic judgements in deluded and non-deluded subjects. *The Quarterly Journal of Experimental Psychology Section A*, *40*(4), 801–812. https://doi.org/[10.1080/14640748808402300](http://dx.doi.org/10.1080/14640748808402300)

Seal, Marc, Andre Aleman, and Philip McGuire 2004 Compelling Imagery, Unanticipated Speech and Deceptive Memory: Neurocognitive Models of Auditory Verbal Hallucinations in Schizophrenia. Cognitive Neuropsychiatry 9(1–2): 43–72

Sullivan, S., Bentall, R. P., Fernyhough, C., Pearson, R. M., & Zammit, S. (2013). Cognitive Styles and Psychotic Experiences in a Community Sample. *PLOS ONE*, *8*(11), e80055. https://doi.org/[10.1371/journal.pone.0080055](http://dx.doi.org/10.1371/journal.pone.0080055)

van der Gaag, M., Schütz, C., ten Napel, A., Landa, Y., Delespaul, P., Bak, M., … de Hert, M. (2013). Development of the Davos Assessment of Cognitive Biases Scale (DACOBS). *Schizophrenia Research*, *144*(1-3), 63–71. https://doi.org/[10.1016/j.schres.2012.12.010](http://dx.doi.org/10.1016/j.schres.2012.12.010)

Waechter, S., Nelson, A. L., Wright, C., Hyatt, A., & Oakman, J. (2014). Measuring Attentional Bias to Threat: Reliability of Dot Probe and Eye Movement Indices. *Cognitive Therapy and Research*, *38*(3), 313–333. https://doi.org/[10.1007/s10608-013-9588-2](http://dx.doi.org/10.1007/s10608-013-9588-2)

Woodward, T. S., Moritz, S., Menon, M., & Klinge, R. (2008). Belief inflexibility in schizophrenia. *Cognitive Neuropsychiatry*, *13*(3), 267–277. https://doi.org/[10.1080/13546800802099033](http://dx.doi.org/10.1080/13546800802099033)

# Search Terms

**Psychinfo search 15/2/20**

1. (JTC or (Jumping adj2 conclusions) or (jump adj2 conclusion) or (data adj gathering) or (beads adj2 task) or probab* reas*).ti,ab,id,tm.

2. (bias adj2 (cognition or cognitive or attention* or process* or perception or perceptual or cognition)).ti,ab,id,tm.

3. (trauma* or maltreat* or abuse or advers* or neglect or bully* or victim* or rape or violen* or assault*).ti,ab,id,tm.

4. ((source adj memory) or (source adj monitoring) or (reality adj monitoring) or (reality adj discrimination) or (source adj recognition) or (external adj monitoring) or (external adj misattribut*) or (internal adj attribut*)).ti,ab,id,tm.

5. "locus of control".ti,ab,id,tm.

6. ((attribut* adj style) or (external adj attribut*) or (attribut* adj style) or (externalis* adj bias) or (externaliz* adj bias)).ti,ab,id,tm.

7. (belief inflex* or BADE or bias against disconfirmatory evidence or disconfirm* bias* or evidence integrat*).ti,ab,id,tm.

8. ((Top-down adj2 process*) or (top adj down adj2 process*) or (auditory adj feedback) or (visual adj feedback)).ti,ab,id,tm.

9. 1 and 3

10. 2 and 3

11. 3 and 4

12. 3 and 5

13. 3 and 6

14. 3 and 7

15. 3 and 8

**Ovid Medliner search 1946 to 15/2/20**

1. (trauma* or maltreat* or abuse or advers* or neglect or bully* or victim* or rape or violen* or assault*).ti,ab,kf.

2. (bias adj2 (cognition or cognitive or attention* or process* or perception or perceptual or cognition)).ti,ab,kf.

3. (JTC or (Jumping adj2 conclusions) or (jump adj2 conclusion) or (data adj gathering) or (beads adj2 task) or probab* reas*).ti,ab,kf.

4. ((source adj memory) or (source adj monitoring) or (reality adj monitoring) or (reality adj discrimination) or (source adj recognition) or (external adj monitoring) or (external adj misattribut*) or (internal adj attribut*)).ti,ab,kf.

5. "locus of control".ti,ab,kf.

6. ((external adj attribut*) or (attribut* adj style) or (externalis* adj bias) or (externaliz* adj bias)).ti,ab,kf.

7. ((Top-down adj2 process*) or (top adj down adj2 process*) or (auditory adj feedback) or (visual adj feedback)).ti,ab,kf.

8. 1 and 2

9. 1 and 3

10. 1 and 4

11. 1 and 5

12. 1 and 6

13. 1 and 7

**PILOTS search 15/2/20**

[(((Top-down NEAR/2 process*) OR (top down NEAR/2 process*) OR (auditory NEAR/1 feedback) OR (visual NEAR/1 feedback)) AND (trauma* OR maltreat* OR abuse OR advers* OR neglect OR bully* OR victim* OR rape OR violen* OR assault*)) AND stype.exact("Scholarly Journals")](https://search.proquest.com/myresearch/savedsearches.checkdbssearchlink:rerunsearch/1293859/SavedSearches?site=pilots&t:ac=SavedSearches)

[((belief NEAR/1 inflex*) OR BADE OR (bias NEAR/1 against NEAR/1 disconfirmatory NEAR/1 evidence) OR (disconfirm* NEAR/1 bias*) OR (evidence NEAR/1 integrat*)) AND (trauma* OR maltreat* OR abuse OR advers* OR neglect OR bully* OR victim* OR rape OR violen* OR assault*) AND stype.exact("Scholarly Journals")](https://search.proquest.com/myresearch/savedsearches.checkdbssearchlink:rerunsearch/1293853/SavedSearches?site=pilots&t:ac=SavedSearches)

[("locus of control" OR ((attribut* NEAR/1 style) OR (external NEAR/1 attribut*) OR (attribut* NEAR/1 style) OR (externalis* NEAR/1 bias) OR (externaliz* NEAR/1 bias))) AND (trauma* OR maltreat* OR abuse OR advers* OR neglect OR bully* OR victim* OR rape OR violen* OR assault*)](https://search.proquest.com/myresearch/savedsearches.checkdbssearchlink:rerunsearch/1293816/SavedSearches?site=pilots&t:ac=SavedSearches)

[((source NEAR/1 memory) OR (source NEAR/1 monitoring) OR (reality NEAR/1 monitoring) OR (reality NEAR/1 discrimination) OR (source NEAR/1 recognition) OR (external NEAR/1 monitoring) OR (external NEAR/1 misattribut*) OR (internal NEAR/1 attribut*)) AND (trauma* OR maltreat* OR abuse OR advers* OR neglect OR bully* OR victim* OR rape OR violen* OR assault*) AND stype.exact("Scholarly Journals")](https://search.proquest.com/myresearch/savedsearches.checkdbssearchlink:rerunsearch/1293800/SavedSearches?site=pilots&t:ac=SavedSearches)

[((JTC) OR (Jumping NEAR/2 conclusions) OR (jump NEAR/2 conclusion) OR (data NEAR/2 gathering) OR (beads NEAR/2 task) OR (probab* reas*)) AND (trauma* OR maltreat* OR abuse OR advers* OR neglect OR bully* OR victim* OR rape OR violen* OR assault*) AND stype.exact("Scholarly Journals")](https://search.proquest.com/myresearch/savedsearches.checkdbssearchlink:rerunsearch/1293814/SavedSearches?site=pilots&t:ac=SavedSearches)

# Fig. A Screening Checklist

|  |  |
| --- | --- |
| Is the paper published in a peer-reviewed journal? | Y/N |
| Does the paper include a measure of trauma or childhood adversity?  Trauma/adversity should refer to:   - Physical abuse - Emotional abuse - Sexual Abuse - Neglect - Bullying   And any exposures where the person was exposed to: death, threatened death, actual or threatened serious injury, or actual or threatened sexual violence.  Stressful life events such as parental divorce / economic adversity, and other events that do not fit in criteria above are not to be included | Y/N |
| Trauma reports must include measures of trauma that have occurred prior to the age of 18 years old | Y/N |
| Does the study measure a cognitive bias listed in the protocol **or** specifically state that it measures a cognitive bias associated with psychosis? | Y/N |
| Does the study compare groups of participants that do and do not report childhood trauma/adversity on a cognitive bias task | Y/N |

# Fig. B Adapted Newcastle-Ottawa Quality Assessment

1) Selection of Participants

a) Reported as randomly or consecutively (completely) sampled*

b) Reported as sampled using convenience sampling

c) No description or unclear description of recruitment method

2) Selection of the non-exposed cohort

a) Drawn from the same community and representative of the exposed cohort*

b) Drawn from a different source

c) No description of the derivation of the non-exposed cohort

3) Selection of recruited participants

a) Response rate of over 75% from selected participant reported*

b) No reported response rate

4) Comparability

a) The study controls for two of the following factors: sex, SES, IQ, family history of psychopathology **

b) The study controls for one of the factors above*

c) No adjustment for any of the confounders listed above

5) Assessment of outcome

a) Independent assessment blind to trauma status *

b) Self report*

c) No description/ other

# Fig. C Funnel plot of Egger’s Test Distribution with pseudo-95% confidence intervals for the association between Childhood Trauma and Locus of Control


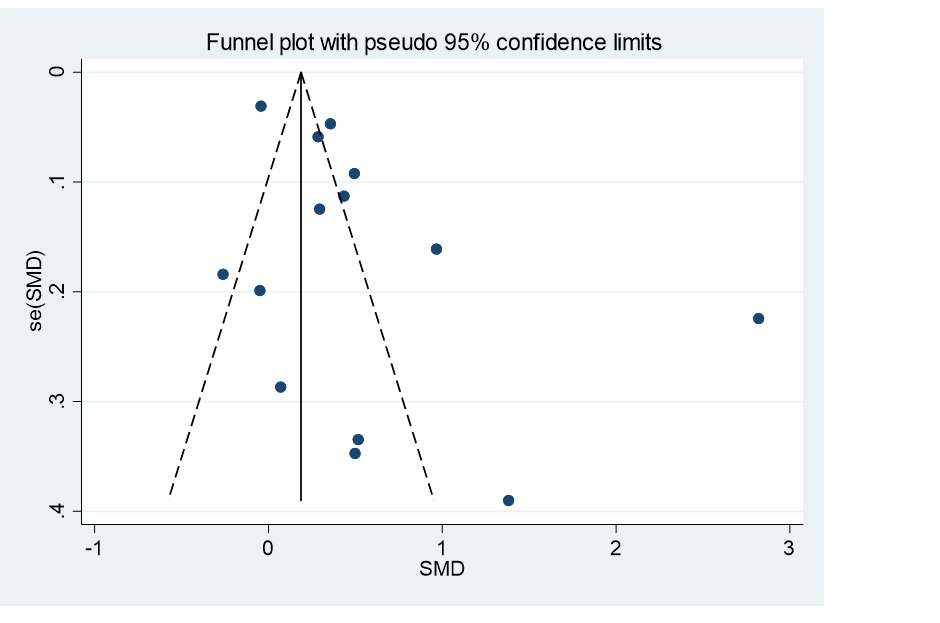


Figure 4.4 Funnel Plot of Distribution of Effects of Childhood Trauma and Locus of Control Meta-Analysis

# Table A Methods of Trauma Assessment in Studies Included in the Review

| **Study** | **Trauma Type** | **Trauma Measure** | **Scale** |
| --- | --- | --- | --- |
| Allen et al., 2017 | Multiple | Questionnaire | Questions in PTSD section of adult co-morbidity survey |
| Andreou, 2000 | Bullying | Questionnaire | Bullying‐Behaviour Scale and the Peer‐Victimisation Scale(Austin and Joseph, 1996) |
| Asberg & Renk, 2014 | Sexual Abuse | Questionnaire | Life events checklist |
| Atik & Guneri, 2013 | Bullying | Questionnaire | Revised Olweus Bully/Victim Questionnaire (OBVQ) (Gonçalves et al., 2016) |
| Barahal, Waterman, & Martin, 1981 | Multiple | Referrals from social services | N/A |
| Beck -Sander et al, 1997 | Sexual Abuse, Physical Abuse | Questionnaire | Sex Events Questionnaire (adapted) (Calam and Slade, 1989) and Physical abuse record (Andrews and Brown, 1988) |
| Bendall et al., 2011 | Multiple | Questionnaire | Childhood Trauma Questionnaire (Bernstein et al., 1997) |
| Bolstad et al., 1997 | Sexual Abuse | Questionnaire | Self-report measure developed by study authors |
| Chiu et al., 2016 | Multiple | Questionnaire | The Brief Betrayal Trauma Survey (Goldberg and Freyd, 2006) |
| Chiu et al., 2018 | Multiple | Questionnaire | The Brief Betrayal Trauma Survey (Goldberg and Freyd, 2006) |
| Fredstrom, Adams, & Gilman, 2011 | Bullying | Questionnaire | Self-report measure developed by study authors |
| Freeman, Pugh, & Garety, 2008 | Multiple | Questionnaire | Life stressor checklist - child trauma section(Wolfe, J., Kimerling, R., 1997) |
| Hovens, Giltay, van Hemert, & Penninx, 2016 | Multiple | Questionnaire | NEMESIS childhood trauma interview(Hovens et al., 2012) |
| Ireland, Alderson, & Ireland, 2015 | Sexual Abuse | Questionnaire | Self-report measure developed by study authors |
| Luciano & Savage, 2007 | Bullying | Questionnaire | The My Life in School checklist(S. Sharp, 1994) |
| Mannarino et al., 1996 | Sexual Abuse | Referrals from social services | N/A |
| Mcnally 2006 | Sexual Abuse | Semi-structured interview | N/A |
| Marsh et al., 2011 | Bullying | Questionnaire | Adolescent peer relations (APRI) developed for study |
| Moran & Eckenrode, 1992 | Multiple | Questionnaire | NR |
| Moyer at al | Sexual Abuse | Referrals from social services | N/A |
| Muller 1994 | Physical Abuse | Questionnaire | Conflict Tactic Scale(Murray Straus and Christine Smith, 1990) |
| Porter & Long, 1999 | Sexual Abuse | Questionnaire | Life experiences questionnaire (developed by study authors) |
| Radliff, Wang, & Swearer, 2016 | Bullying | Questionnaire | The Verbal and Physical Bullying Scale–Victimization scale (Swearer et al., 2008) |
| Roazzi et al, 2016 | Multiple | Referrals from social services | N/A |
| Rucklidge, 2006 | Multiple | Questionnaire | Childhood Trauma Questionnaire(Bernstein et al., 1997) |
| Yamasaki et al | Bullying | Questionnaire | Olweus Bully/Victim Questionnaire(Solberg and Olweus, 2003) |

NOTE NR = Not Reported

# Table B Meta-Regression of Pooled Analysis of Association Between Childhood Trauma and Locus Of Control

|  | Coefficient | Confidence Intervals | *p* | I^2^ (%) |
| --- | --- | --- | --- | --- |
| Mean Age | -.013 | -.04, .01 | .241 | 88.0 |
| % female | -.002 | -.012, .001 | .537 | 76.3 |
| Recruitment sample* | 0.50 | -.15, .1.15 | .120 | 90.1 |
| Multiple Trauma Types or Single Trauma type | .014 | -58, .61 | .957 | 84.4 |
| Quality Assessment score | -.22 | -.59, 1.4 | .206 | 91.4 |

^a^All analysis excludes one study (Andreou et al) identified as an outlier in the main analysis *Population-based or not population-based

# Table C: Selected studies meeting some, but not all inclusion criteria

| Author (year) | Study Design | Sample | Sample Selection | Main outcome Cognitive Bias Measure | Main Findings | Reason for exclusion |
| --- | --- | --- | --- | --- | --- | --- |
| Thurber et al., (1977) | Observational | 181 women selected at random from six rural mining communities in the Pacific Northwest | Women exposed to fire and floods in region | Locus of control (Rotter’s Internal-External scale) | Women who were evacuated due to effects of flooding compared to those not evacuated reported a more external LOC (t=2.43 p<.02) | Exposure to trauma in adulthood |
| (Rhodes et al., 1993) | Observational | 177 pregnant and parenting African-American adolescent women (M=18.34 years, SD=1.79) | Recruited from local social services | Locus Of Control (Nowicki-Strickland) | Victimised women (n=39) had more external LOC compared to non-victimised (n=138) p<.05 | Exposure to trauma in adulthood |
| (Walsh et al., 2007) | Observational | 73 female undergraduate students 18-27 years old | Recruited via a job university website at a university in USA | Locus of Control (Internality, Powerful Others, Chance) | Women reporting any non-consensual sex in adulthood had a lowered internal LOC (F (1,72) = 5.68, *p<.*0.5) | Exposure to trauma in adulthood |
| (Gawęda et al., 2017) | Observational | 653 undergraduate students 18-27 years old (M=22.24, SD 3.14) | Only students with no reported history of psychiatric or neurological disease included | Davos Assessment of Cognitive Biases (DACOBS): Jumping to Conclusions, Belief Inflexibility, Attention for Threat, External Attribution | Exposure to traumatic events highly correlated with attention to threat and external attributions (<.0001) but not with belief inflexibility or Jumping to Conclusions Bias | Exposure to trauma in both childhood adulthood |

# Bibliography

Aleman, A., Böcker, K.B.E., Hijman, R., de Haan, E.H.F., and Kahn, R.S. (2003). Cognitive basis of hallucinations in schizophrenia: role of top-down information processing. Schizophr. Res. *64*, 175–185.

Allen, B., and Lauterbach, D. (2007). Personality characteristics of adult survivors of childhood trauma. J. Trauma. Stress *20*, 587–595.

Andreou, E. (2000). Bully/victim problems and their association with psychological constructs in 8- to 12-year-old Greek schoolchildren. Spec. Issue Bullying Sch. *26*, 49–56.

Andrews, B., and Brown, G.W. (1988). Marital Violence in the Community: A Biographical Approach. Br. J. Psychiatry *153*, 305–312.

Asberg, K., and Renk, K. (2014). Perceived Stress, External Locus of Control, and Social Support as Predictors of Psychological Adjustment Among Female Inmates With or Without a History of Sexual Abuse. Int. J. Offender Ther. Comp. Criminol. *58*, 59–84.

Atik, G., and Guneri, O.Y. (2013). Bullying and victimization: Predictive role of individual, parental, and academic factors. Sch. Psychol. Int. *34*, 658–673.

Austin, S., and Joseph, S. (1996). Assessment of bully/victim problems in 8 to 11 year-olds. Br. J. Educ. Psychol. *66*, 447–456.

Barahal, R.M., Waterman, J., and Martin, H.P. (1981). The social cognitive development of abused children. J. Consult. Clin. Psychol. *49*, 508–516.

Beck-sander, A. (1995). Childhood Abuse in Adult Offenders: The Role of Control in Perpetuating Cycles of Abuse. J. Forensic Psychiatry *6*, 486–498.

Bendall, S., Jackson, H.J., and Hulbert, C.A. (2011). What self-generated speech is externally misattributed in psychosis? Testing three cognitive models in a first-episode sample. Schizophr. Res. *129*, 36–41.

Bernstein, D.P., Ahluvalia, T., Pogge, D., and Handelsman, L. (1997). Validity of the Childhood Trauma Questionnaire in an adolescent psychiatric population. J. Am. Acad. Child Adolesc. Psychiatry *36*, 340–348.

Bolstad, B.R., and Zinbarg, R.E. (1997). Sexual victimization, generalized perception of control, and posttraumatic stress disorder symptom severity. J. Anxiety Disord. *11*, 523–540.

Calam, R.M., and Slade, P.D. (1989). Sexual experience and eating problems in female undergraduates. Int. J. Eat. Disord. *8*, 391–397.

Chiu, C.-D., Tseng, M.-C.M., Chien, Y.-L., Liao, S.-C., Liu, C.-M., Yeh, Y.-Y., and Hwu, H.-G. (2016). Misattributing the Source of Self-Generated Representations Related to Dissociative and Psychotic Symptoms. Front. Psychol. *7*, 541.

Choi, K.H., Davidson, C., and Spaulding, W.D. (2011). Social cognition moderates the influence of child physical abuse on inpatient psychiatric rehabilitation. J. Nerv. Ment. Dis. *199*, 465–470.

Fredstrom, B.K., Adams, R.E., and Gilman, R. (2011). Electronic and school-based victimization: unique contexts for adjustment difficulties during adolescence. J. Youth Adolesc. *40*, 405–415.

Freeman, D., Pugh, K., and Garety, P. (2008). Jumping to conclusions and paranoid ideation in the general population. Schizophr. Res. *102*, 254–260.

Gawęda, Ł., Prochwicz, K., Adamczyk, P., Frydecka, D., Misiak, B., Kotowicz, K., Szczepanowski, R., Florkowski, M., and Nelson, B. (2017). The role of self-disturbances and cognitive biases in the relationship between traumatic life events and psychosis proneness in a non-clinical sample. Schizophr. Res.

Goldberg, L.R., and Freyd, J.J. (2006). Self-reports of potentially traumatic experiences in an adult community sample: gender differences and test-retest stabilities of the items in a brief betrayal-trauma survey. J. Trauma Dissociation Off. J. Int. Soc. Study Dissociation ISSD *7*, 39–63.

Gonçalves, F.G., Heldt, E., Peixoto, B.N., Rodrigues, G.A., Filipetto, M., and Guimarães, L.S.P. (2016). Construct validity and reliability of Olweus Bully/Victim Questionnaire – Brazilian version. Psicol. Reflex. E Crítica *29*, 27.

Hovens, J.G.F.M., Giltay, E.J., Wiersma, J.E., Spinhoven, P., Penninx, B.W.J.H., and Zitman, F.G. (2012). Impact of childhood life events and trauma on the course of depressive and anxiety disorders. Acta Psychiatr. Scand. *126*, 198–207.

Hovens, J.G.F.M., Giltay, E.J., van Hemert, A.M., and Penninx, B.W.J.H. (2016). CHILDHOOD MALTREATMENT AND THE COURSE OF DEPRESSIVE AND ANXIETY DISORDERS: THE CONTRIBUTION OF PERSONALITY CHARACTERISTICS. Depress. Anxiety *33*, 27–34.

Kaney, S., and Bentall, R.P. (1989). Persecutory delusions and attributional style. Br. J. Med. Psychol. *62*, 191–198.

Luciano, S., and Savage, R.S. (2007). Bullying risk in children with learning difficulties in inclusive educational settings. Can. J. Sch. Psychol. *22*, 14–31.

MANNARINO, A.P., and COHEN, J.A. (1996). Abuse-Related Attributions and Perceptions, General Attributions, and Locus of Control in Sexually Abused Girls , Abuse-Related Attributions and Perceptions, General Attributions, and Locus of Control in Sexually Abused Girls. J. Interpers. Violence *11*, 162–180.

Marsh, H.W., Nagengast, B., Morin, A., J. S., re, Parada, R.H., Craven, R.G., and Hamilton, L.R. (2011). Construct validity of the multidimensional structure of bullying and victimization: An application of exploratory structural equation modeling. J. Educ. Psychol. *103*, 701–732.

McNally, R.J., Clancy, S.A., Barrett, H.M., and Parker, H.A. (2005). Reality monitoring in adults reporting repressed, recovered, or continuous memories of childhood sexual abuse. J. Abnorm. Psychol. *114*, 147–152.

Moran, P.B., and Eckenrode, J. (1992). Protective personality characteristics among adolescent victims of maltreatment. Child Abuse Negl. *16*, 743–754.

Moyer, D.M., DiPietro, L., Berkowitz, R.I., and Stunkard, A.J. (1997). Childhood sexual abuse and precursors of binge eating in an adolescent female population. Int. J. Eat. Disord. *21*, 23–30.

Muller, R.T., Caldwell, R.A., and Hunter, J.E. (1994). Factors predicting the blaming of victims of physical child abuse or rape. Can. J. Behav. Sci. Rev. Can. Sci. Comport. *26*, 259–279.

Murray Straus, R.G., and Christine Smith (1990). Physical violence in American families: Risk factors and adaptations to violence in 8,145 families (New Brunswick, NJ: Transaction).

Porter, C.A., and Long, P.J. (1999). Locus of control and adjustment in female adult survivors of childhood sexual abuse. J. Child Sex. Abuse Res. Treat. Program Innov. Vict. Surviv. Offenders *8*, 3–25.

Radliff, K.M., Wang, C., and Swearer, S.M. (2016). Bullying and Peer Victimization: An Examination of Cognitive and Psychosocial Constructs. J. Interpers. Violence *31*, 1983–2005.

Rhodes, J.E., Fischer, K., Ebert, L., and Meyers, A.B. (1993). Patterns of service utilization among pregnant and parenting African American adolescents. Psychol. Women Q. *17*, 257–274.

Roazzi, A., Attili, G., Pentima, L.D., and Toni, A. (2016). Locus of control in maltreated children: the impact of attachment and cumulative trauma. Psicol. Reflex. E Crítica *29*, 8.

Rucklidge, J.J. (2006). Psychosocial functioning of adolescents with and without paediatric bipolar disorder. J. Affect. Disord. *91*, 181–188.

S. Sharp, P.K.S. (1994). Understanding bullying. In Tackling Bullying in Your School: A Practical Handbook for Teachers, (Routledge), p.

Seal, M., Aleman, A., and McGuire, P. (2004). Compelling imagery, unanticipated speech and deceptive memory: Neurocognitive models of auditory verbal hallucinations in schizophrenia. Cognit. Neuropsychiatry *9*, 43–72.

Solberg, M.E., and Olweus, D. (2003). Prevalence estimation of school bullying with the Olweus Bully/Victim Questionnaire. Aggress. Behav. *29*, 239–268.

Sullivan, S., Bentall, R.P., Fernyhough, C., Pearson, R.M., and Zammit, S. (2013). Cognitive Styles and Psychotic Experiences in a Community Sample. PLOS ONE *8*, e80055.

Swearer, S.M., Turner, R.K., Givens, J.E., and Pollack, W.S. (2008). “You’re so gay!”: Do different forms of bullying matter for adolescent males? Sch. Psychol. Rev. *37*, 160–173.

Walsh, D.K., Blaustein, M., Knight, W.G., Spinazzola, J., and Kolk, B.A. van der (2007). Resiliency Factors in the Relation Between Childhood Sexual Abuse and Adulthood Sexual Assault in College-Age Women. J. Child Sex. Abuse *16*, 1–17.

Wolfe, J., Kimerling, R., (1997). Gender issues in the assessment of posttraumatic stress disorder. In Assessing Psychological Trauma and PTSD., (New York: Guilford Press), p.

Yamasaki, S., Ando, S., Koike, S., Usami, S., Endo, K., French, P., Sasaki, T., Furukawa, T.A., Hasegawa-Hiraiwa, M., Kasai, K., et al. (2016). Dissociation mediates the relationship between peer victimization and hallucinatory experiences among early adolescents. Schizophr. Res. Cogn. *4*, 18–23.

Irel, Carol A., Alderson, K., Irel, and Jane L. (2015). Sexual exploitation in children: Nature, prevalence, and distinguishing characteristics reported in young adulthood. J. Aggress. Maltreatment Trauma *24*, 603–622.
